# Supplementary material for: Associations between Dietary Patterns and Cardiometabolic Risk Factors—A Longitudinal Analysis among High-Risk Individuals for Diabetes in Kerala, India
Source: Nutrients. 2022 Feb 4;14(3):662. doi: 10.3390/nu14030662 (PMC8838960; doi:10.3390/nu14030662)
Supplement: Supplementary file 1 [file nutrients-14-00662-s001.zip › nutrients-1539786-supplementary.pdf]

Supplementary Table S1. K-DPP FOOD FREQUENCY QUESTIONNAIRE

Code	Food	No. of times/day	If not daily then No. of times/week	If not weekly then No. of times/month	Never	Portion size	Number of servings/pieces per meal
**1. Rice based products**							
1.1	Boiled rice<br>What type of rice do you eat?<br>_____________					Small<br>Medium<br>Large<br>> largest portion	Of what size?
1.2	Rice gruel						
1.3	Idly						
1.4	Dosa						
1.5	Puttu						
1.6	Appam						
1.7	Idiyappam						
1.8	Other (specify)						

Code	Food	No. of times/day	If not daily then No. of times/week	If not weekly then No. of times/month	Never	Number of servings/pieces per meal
**2. Wheat based foods**						
2.1	Chapathy					
2.2	Puris					
2.3	Roti					
2.4	Parratas					
2.5	Wheat puttu					
2.6	Other (specify)					

Code	Food	No. of times/day	If not daily then No. of times/week	If not weekly then No. of times/month	Never	Number of servings/pieces per meal
**3. Legumes and Pulses**						
3.1	Lentils (broken dal, black gram, green gram etc)					
3.2	Bengal gram					

3.3	Peanuts					
3.4	Other (e.g. kidney beans)					

Code	Food	No. of times/day	If not daily then No. of times/week	If not weekly then No. of times/month	Never	Number of servings/pieces per meal
**4. Coconut**						
4.1	Tender coconut					
4.2	Tender coconut water					
4.3	Grated coconut					
4.4	Other (specify)					

Code	Food	No. of times/day	If not daily then No. of times/week	If not weekly then No. of times/month	Never	Number of servings/pieces per meal
**5. Vegetables**						

5.1	Root tuber vegetables (e.g. Yam, katchil, koorkha, colocasia, beetroot, potato, sweet potato, pumpkin, casava)					
5.2	Pumpkin					
5.3	Vegetables (e.g. chiqur manis, brinjal, carrot cucumber, tomato, cauliflower, green beans, peas, etc.)					What size cup?<br>40grams<br>80grams
5.4	Other (specify)					

Code	Food	No. of times/day	If not daily then No. of times/week	If not weekly then No. of times/month	Never	Number of servings/pieces per meal
**6. Fruits**						
6.1	Banana (small, medium, large)					
6.2	Papaya					
6.3	Mango					
6.4	Jackfruit (pieces)					
6.5	Apple					

6.6	Grapes					
6.7	Fruit juice					
6.8	Other (specify)					

Code	Food	No. of times/day	If not daily then No. of times/week	If not weekly then No. of times/month	Never	Number of servings/pieces per meal
**7. Cooking oil**						
7.1	Coconut oil					
7.2	Ghee					
7.3	Dalda					
7.4	Palm oil					
7.5	Other (e.g. peanut oil, mustard oil, sunflower oil)					

Code	Food	No. of times/day	If not daily then No. of times/week	If not weekly then No. of times/month	Never	Number of servings/pieces per meal

**8. Sweet items**						
8.1	Sugary drinks (e.g. non-diet carbonated soft drinks, tea, cordials, lassi)					
8.2	Cakes and pastries (including puff)					
8.3	Biscuits					
8.4	Mithai					
8.5	Sweet parotta					
8.6	Other (specify)					

Code	Food	No. of times/day	If not daily then No. of times/week	If not weekly then No. of times/month	Never	Number of servings/pieces per meal
**9. Fried foods and snacks**						
9.1	Fried chips (banana, potato, jackfruit, tapioca)					
9.2	Vada and pakoda					
9.3	Murukku					
9.4	Mixture					

9.5	Papad					
9.6	Other (specify)					

Code	Food	No. of times/day	If not daily then No. of times/week	If not weekly then No. of times/month	Never	Number of servings/pieces per meal
**10. Meat, fish and eggs**						
11.1	Fish(including dried)					
11.2	Beef					
11.3	Chicken					
11.4	Eggs					
11.5	Other (e.g mutton, pork etc.					

Respondent: 1: Head of household/spouse 2. Any other (specify)_____________________

Name of interviewer: _______________________________________

Date: _______________________ Signature: _________________________

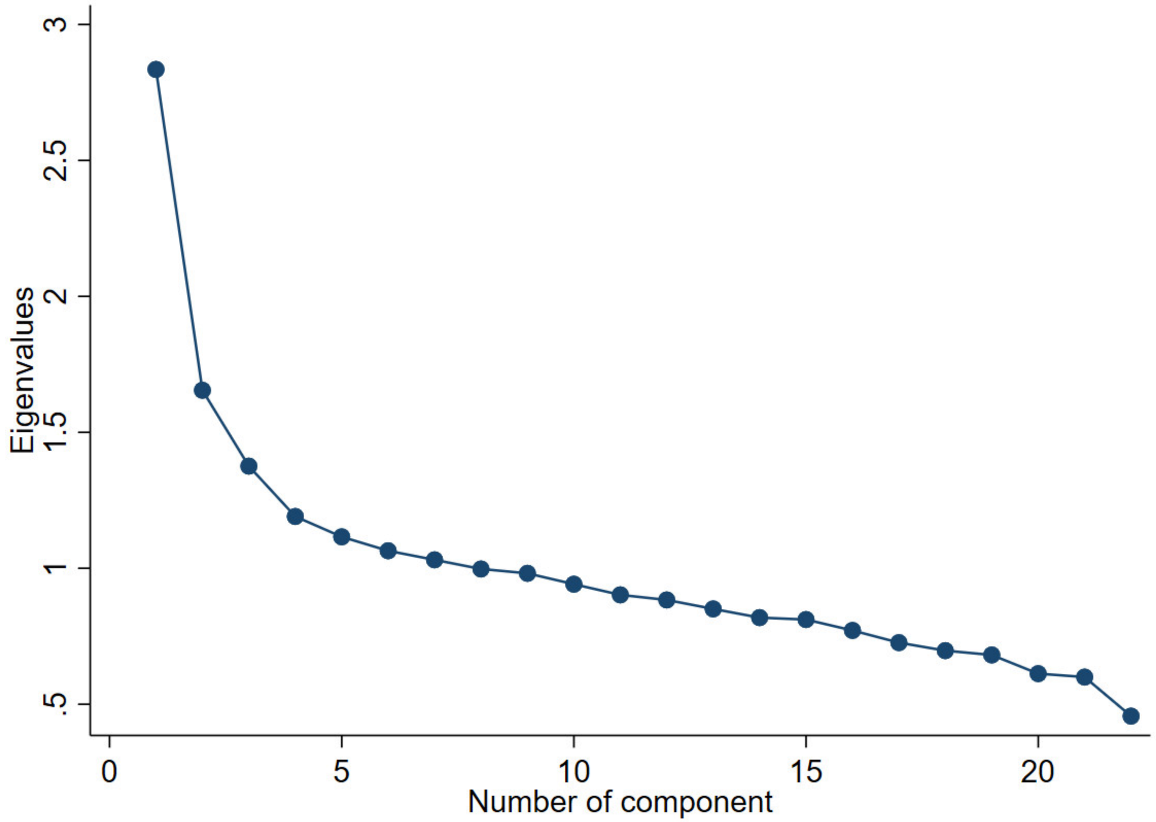

Supplementary Figure S1. Scree plot for Principal component analysis

It displays the eigenvalues of factors or principal components on Y-axis and the number of potential components (factors) on X axis from the PCA analysis. The point where the slope of the curve is clearly leveling off (the “elbow”) indicates the number of factors that should be generated by the analysis.
